# Altered dendritic cell distribution in patients with common variable immunodeficiency

**DOI:** 10.1186/ar1774

**Published:** 2005-07-01

**Authors:** Jean-François Viallard, Fabrice Camou, Marc André, François Liferman, Jean-François Moreau, Jean-Luc Pellegrin, Patrick Blanco

**Affiliations:** 1 Department of Internal Medicine and Infectious Diseases, Haut-Lévêque Hospital, Pessac, France; 2 Department of Internal Medicine, Gabriel-Montpied Hospital, Clermont-Ferrand, France; 3 Department of Internal Medicine, Dax Hospital, Dax, France; 4 Laboratory of Immunology, Pellegrin Hospital, Bordeaux, France

## Abstract

Recent data suggest a critical role for dendritic cells (DCs) in the generation of immunoglobulin-secreting plasma cells. In the work reported herein, we analyzed the frequency of peripheral blood plasmacytoid DCs (pDCs) and myeloid DCs (mDCs) in a cohort of 44 adults with common variable immunodeficiency (CVID) classified according to their CD27 membrane expression status on B cells. A deep alteration in the distribution of DC subsets, especially of pDCs, in the peripheral blood of CVID patients was found. Patients with a reduced number of class-switched CD27^+^IgD^-^IgM^- ^memory B cells and patients with granulomatous disease had a dramatic decrease in pDCs (*P * = 0.00005 and 0.0003 vs controls, respectively) and, to a lesser extent, of mDCs (*P * = 0.001 and 0.01 vs controls, respectively). In contrast, patients with normal numbers of switched memory B cells had a DC distribution pattern similar to that in controls. Taken together, our results raise the possibility that innate immunity contributes to pathogenesis in CVID.

## Introduction

Common variable immunodeficiency (CVID) is a primary immunodeficiency disease characterized by a low concentration of serum immunoglobulins and an impaired antibody response to challenging antigens [1]. Although the pathophysiology of CVID is heterogeneous and largely unknown, several causes leading to an alteration of immunoglobulin concentrations in the blood have already been identified. These include a failure of B-cell maturation, including altered somatic hypermutation [2]; defective cell-membrane signaling [3]; T-cell abnormalities such as reduced expression of key membrane-expressed molecules (CD40 ligand, ICOS (inducible costimulator protein), L-selectin) [4]; impaired cytokine production [5]; and a reduced generation of antigen-specific memory T cells [6].

Whereas the antigen-presenting function of DCs has been reported to be normal in CVID [7,8], their involvement in the origin of some CVID cannot be ruled out, as these cells are known to interact directly with B cells, to present antigen to T cells, and to produce cytokines implicated in B-cell differentiation [9]. Two major pathways of differentiation generating DCs are thought to exist, according to their membrane expression of the β-integrin CD11c [10]. Myeloid DCs (mDCs) include skin Langerhans' cells and interstitial DCs and express CD11c at their surface. In contrast, plasmacytoid DCs (pDCs), which do not express CD11c, are CD123^+^. Recent data suggest a role for DCs in B-cell growth and differentiation, as the release by mDCs of soluble factors such as IL-12 and IL-6 and/or membrane molecules such as BAFF/APRIL induces the activation and the differentiation of normal B cells [11,12]. In addition, the observation that pDCs directly induce the differentiation of plasma cells into immunoglobulin-secreting plasma cells suggests that pDCs are critically involved in humoral responses [13,14]. Altogether, these new data prompted us to examine the blood distribution of DC subsets in 44 patients with CVID.

## Materials and methods

### Patient characteristics

Forty-four patients with CVID (17 to 77 years of age; 28 women and 17 men) were enrolled in this study after they had given their informed consent and following the approval of the local Ethics Committee. All patients were diagnosed as having CVID, on the basis of a specific medical history of recurrent bacterial infections associated with hypogammaglobulinemia (serum immunoglobulin (Ig)G and IgA and/or IgM at least two standard deviations below the normal mean) [15]. At the time of evaluation, none of the patients showed evidence of acute infection. As is frequently observed in CVID, 13 of the 45 patients had autoimmune diseases, 14 had splenomegaly, 5 had lymphoid hyperplasia, and 7 presented with a chronic granulomatous disease.

All the patients included in this study had blood CD19^+^CD3^- ^B-lymphocyte counts above 1% of peripheral blood lymphocytes. The CVID patients were divided into two groups according to the detection of switched memory B cells (CD27^+^IgD^-^) as recently proposed by Warnatz and colleagues [16]. Group 1 (*n * = 22), comprising patients whose proportion of switched memory B cells was less than 0.4% of their total peripheral blood lymphocytes, was further subdivided according to whether they had increased (group 1a; *n * = 13) or normal (group 1b; *n * = 9) numbers of CD19^+^CD21^- ^immature B cells. Group 2 (*n * = 15) comprised patients whose proportion of switched memory B cells was more than 0.4% of total peripheral blood lymphocytes. As Warnatz and colleagues excluded from their classification patients with granulomatous disease, we classified these patients in a distinct group (group 3; *n * = 7). Control patients were healthy Caucasian blood donors and health-care workers (*n * = 12, median 36 years; 8 women and 4 men); they were not matched with patients for sex or age.

### Quantitation of blood DC precursors by flow cytometry

DC subsets were measured using the DC kit purchased from BD Biosciences (Pont-de-Claix, France). Samples were analyzed on a FACSCalibur flow cytometer (BD Biosciences) and 10^6 ^white blood cells were acquired. Peripheral blood mDC and pDC subsets were defined by the concomitant lack of lineage markers, HLA-DR expression, and mutually exclusive membrane expression of CD11c or CD123, respectively. Absolute numbers of blood DC precursor subsets were calculated as percentage of white blood cells or expressed per ml of peripheral blood. Membrane expression of chemokine receptors was assessed by flow cytometry using a triple combination of monoclonal antibodies: BDCA2-FITC (Miltenyi Biotec, Paris, France); and HLA-DR-PerCp; and CCR2-PE or CCR5-PE or CCR7-PE or CXCR3-PE or CXCR4-PE (BD Biosciences).

### Statistical analysis

Median values found for blood cells were compared between groups using the nonparametric Mann–Whitney *U * test, with a level of significance of *P * = 0.05. The Spearman test was used to make correlations. The tests were performed with the statistical software Statistica Inc (Statsoft, Tucson, AZ, USA).

## Results and discussion

**Figure 1:**
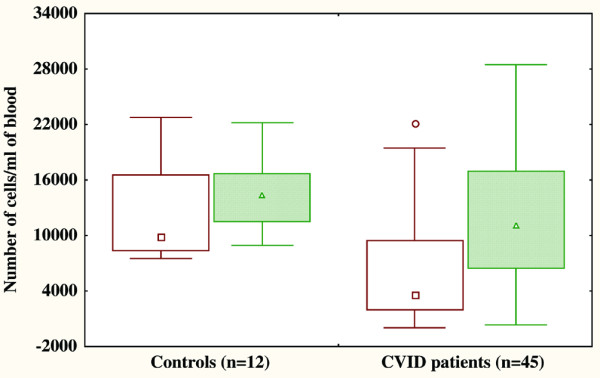
Absolute counts of pDCs and mDCs in 45 CVID patients and 12 controls. Medians of absolute counts of peripheral blood lymphoid dendritic cells (pDCs) (white boxes; □∘) and myeloid DCs (shaded boxes; Δ) in 12 control subjects (respective median values, 9,829/ml and 14,361/ml; extreme values, 7,514 to 22,769/ml and 8,941 to 22,204/ml) and 44 patients with common variable immunodeficiency (CVID) (respective median values, 3,647/ml and 11,100/ml; extreme values, 19 to 22,144/ml and 342 to 28,472/ml). The symbol within each box represents the median value. The upper and lower edges of the boxes represent the 25th and 75th percentiles, and the bars on the whiskers represent the 10th and 90th percentiles. s represents an aberrant value. Statistical comparisons between the two populations (nonparametric Mann–Whitney *U * test): *P * = 0.002 for pDCs and *P * = 0.1 for mDCs.

The median absolute count of circulating pDCs was significantly lower in CVID patients than in controls (*P * = 0.002). Although CVID patients had a lower mean mDC count, their median absolute count was not statistically significantly different from that of controls (*P * = 0.1) (Fig. 1).

However, when CVID patients were segregated in accordance with the Warnatz classification, the DC subset distributions were quite different between groups (Table 1). Groups 1a and 3 had statistically significantly lower levels of circulating DCs of both types than controls, but this difference was far more dramatic concerning the pDC subset (*P * = 0.001 and *P * = 0.01 for mDCs and *P * = 0.00005 and 0.0003 for pDCs, respectively). In groups 1b and 2, blood pDCs and mDCs were not statistically different in comparison with those in controls.

When the various groups of CVID patients were compared with each other, the median absolute count of circulating mDCs was found to be significantly reduced in groups 1a and 3 vs group 2 (*P * = 0.009 and *P * = 0.02, respectively). Although the median values of mDCs were lower in group 1a than in group 1b, the difference did not reach statistical significance (*P * = 0.1). In contrast, CVID patients in groups 1a and 3 had significantly lower pDCs median counts than those in group 2 (*P * = 0.0005 and *P * = 0.001, respectively), and, to a lesser extent, in group 1b (*P * = 0.02 and *P * = 0.009). No differences were observed between group 1a and group 3.

Moreover, we found a significant inverse statistical correlation between the low peripheral pDC levels of 19 CVID patients and the expression of the chemokine receptor CCR7 (*R * = -2.31, *P * = 0.03, Spearman test). These 19 patients were followed in the Department of Internal Medicine at Haut-Lévêque Hospital, directed by JL Pellegrin and JF Viallard. They were chosen because blood samples could easily be taken, in consideration of the short time-period available to perform new experiments with chemokine receptors. Of these 19 patients, 5 belonged to group Ia, 5 to group Ib, 6 to group II and 3 to group III. No differences were observed between the different groups with regard to the expression of other chemokine receptors including CCR2, CCR5, CXCR3, and CXCR4. This observation is in agreement with the already published data concerning the CCL19- and CCL21-dependent migration of mature pDCs to lymph nodes [17].

The most salient feature of the present study concerns patients in group 1a, who were at higher risk of autoimmune diseases (10 of 13 patients) and splenomegaly (9 of 13), and in group 3, both groups exhibiting a marked decrease in their peripheral pDC levels. Patients in group 2, who did not develop such complications, were similar to healthy individuals.

From these preliminary observations we can only speculate on the diminished pDC counts observed in the patients of groups 1a and 3. The migratory properties of DCs seem to be altered, leading to sequestration in tissue and/or secondary lymphoid organs due to an alteration of chemokine receptor membrane expression and maturation. However, we cannot rule out a defect in their function, such as has been suggested regarding DCs derived *in vitro * from monocytes [18].

At least in some patients, hypogammaglobulinemia could rely on an alteration of the innate immunity, since pDCs seem to play a critical role in the generation of antibody responses: they have been shown *in vitro * to control the differentiation of activated B cells into plasma cells through the secretion of interferon α and β and of IL-6 [13]. B cells activated with pDCs preferentially secrete IgG over IgM, suggesting that pDCs may specifically target memory B cells, whereas mDCs, which can enhance B-cell proliferation and isotype switching toward IgA [19], as well as plasma cell differentiation [20], could induce the differentiation of naive B cells through the secretion of IL-12 and IL-6 [11]. Granulocyte-colony-stimulating factor and FLT3 ligand, through their ability to mobilize dendritic cells, may be a new and valuable therapeutic alternative for CVID patients.

## Conclusion

Our data provide evidence of a profound alteration in the distribution of subsets of DCs, especially pDCs, in the peripheral blood of CVID patients. Taken together, our results raise the possibility that innate immunity is involved in CVID pathogenesis.

## Abbreviations

CVID = common variable immunodeficiency; Ig = immunoglobulin; IL = interleukin; mDC = myeloid dendritic cell; pDC = plasmacytoid dendritic cell.

## Competing interests

The authors declare that they have no competing interests.

## Authors' contributions

JFV and FC were in charge of most of the CVID patients gathered in this study, performed the statistical analysis, analyzed the data, and contributed to the writing of the article. PB put together the data, supervised the laboratory examinations, analyzed the data, and contributed to the writing of the article. JFM analyzed the data, participated in the laboratory examinations, contributed to the editing of the article, and supervised the research group. JLP was, along with JFV, in charge of most of the CVID patients gathered in this study and contributed to the writing of the article. MA and FL were in charge of CVID patients and contributed to the writing and preparation of the article. All authors read and approved the final manuscript.

## Figures and Tables

**Table 1:** Absolute counts of peripheral blood DCs in controls and in patients with CVID

Dendritic cells per ml	Healthy controls (*n * = 12)	CVID patients^a^			
		
		Group 1a (*n * = 13)	Group 1b (*n * = 9)	Group 2 (*n * = 15)	Group 3 (*n * = 7)
Myeloid DCs					

Total (median)	14,361	8,432*,^θ^	13,475	15,267	5,568^Ψ,φ^
Percentiles (25th, 75th)	11,470, 16,693	1,428, 11,330	8,378, 16,143	9,462, 18,590	4,315, 11,100
Range	8,941–22,204	342–21,895	3,039–19,385	6,178–28,472	3,787–22,994

Plasmacytoid DCs					

Total pDCs (median)	9,829	2,367*,^λ,θ^	3,647	8,980	1,403^Ψ,ξ,φ^
Percentiles (25th, 75th)	8,366, 16,555	353, 5,012	2,532, 11,590	4,096, 13,367	661, 2,448
Range	7,514–22,769	19–8,291	2,004–13,714	1,614–25,725	417–4,917

^a^Patients were grouped according to whether their proportion of switched memory B cells was <0.4% (groups 1a, 1b) or >0.4% (group 2) of total peripheral blood lymphocytes or they had granulomatous disease (group 3). Group 1 was subdivided according to whether numbers of CD19^+^CD21^- ^immature B cells were increased (group 1a) or normal (group 1b). *P  < 0.001 vs controls;^θ^*P * < 0.009 vs group 2; ^λ^*P * = 0.02 vs group 1b; ^Ψ^*P * < 0.01 vs controls; ^ξ^*P * < 0.009 vs group 1b; ^φ^*P * < 0.02 vs group 2. Other comparisons between each population were not statistically significant. The nonparametric Mann–Whitney *U * test was used for comparisons. CVID, common variable immunodeficiency.

## References

[B1] Cunningham-Rundles C, Bodian C (1999). Common variable immunodeficiency: clinical and immunological features of 248 patients. Clin Immunol.

[B2] Levy Y, Gupta N, Le Deist F, Garcia C, Fischer A, Weill JC, Reynaud CA (1998). Defect in IgV gene somatic hypermutation in common variable immuno-deficiency syndrome. Proc Natl Acad Sci USA.

[B3] Eisenstein EM, Strober W (1995). Evidence for a generalized signaling abnormality in B cells from patients with common variable immunodeficiency. Adv Exp Med Biol.

[B4] Grimbacher B, Hutloff A, Schlesier M, Glocker E, Warnatz K, Drager R, Eibel H, Fischer B, Schaffer AA, Mages HW (2003). Homozygous loss of ICOS is associated with adult-onset common variable immunodeficiency. Nat Immunol.

[B5] Pastorelli G, Roncarolo MG, Touraine JL, Peronne G, Tovo PA, de Vries JE (1989). Peripheral blood lymphocytes of patients with common variable immunodeficiency (CVI) produce reduced levels of interleukin-4, interleukin-2 and interferon-gamma, but proliferate normally upon activation by mitogens. Clin Exp Immunol.

[B6] Kondratenko I, Amlot PL, Webster AD, Farrant J (1997). Lack of specific antibody response in common variable immunodeficiency (CVID) associated with failure in production of antigen-specific memory T cells. MRC Immunodeficiency Group. Clin Exp Immunol.

[B7] Thon V, Eggenbauer H, Wolf HM, Fischer MB, Litzman J, Lokaj J, Eibl MM (1997). Antigen presentation by common variable immunodeficiency (CVID) B cells and monocytes is unimpaired. Clin Exp Immunol.

[B8] Fischer MB, Hauber I, Wolf HM, Vogel E, Mannhalter JW, Eibl MM (1994). Impaired TCR signal transduction, but normal antigen presentation, in a patient with common variable immunodeficiency. Br J Haematol.

[B9] Banchereau J, Briere F, Caux C, Davoust J, Lebecque S, Liu YJ, Pulendran B, Palucka K (2000). Immunobiology of dendritic cells. Annu Rev Immunol.

[B10] Liu YJ (2001). Dendritic cell subsets and lineages, and their functions in innate and adaptive immunity. Cell.

[B11] Dubois B, Massacrier C, Vanbervliet B, Fayette J, Briere F, Banchereau J, Caux C (1998). Critical role of IL-12 in dendritic cell-induced differentiation of naive B lymphocytes. J Immunol.

[B12] MacLennan I, Vinuesa C (2002). Dendritic cells, BAFF, and APRIL: innate players in adaptive antibody responses. Immunity.

[B13] Jego G, Palucka AK, Blanck JP, Chalouni C, Pascual V, Banchereau J (2003). Plasmacytoid dendritic cells induce plasma cell differentiation through type I interferon and interleukin 6. Immunity.

[B14] Poeck H, Wagner M, Battiany J, Rothenfusser S, Wellisch D, Hornung V, Jahrsdorfer B, Giese T, Endres S, Hartmann G (2004). Plasmacytoid dendritic cells, antigen, and CpG-C license human B cells for plasma cell differentiation and immunoglobulin production in the absence of T-cell help. Blood.

[B15] Conley ME, Notarangelo LD, Etzioni A (1999). Diagnostic criteria for primary immunodeficiencies. Representing PAGID (Pan-American Group for Immunodeficiency) and ESID (European Society for Immunodeficiencies). Clin Immunol.

[B16] Warnatz K, Denz A, Drager R, Braun M, Groth C, Wolff-Vorbeck G, Eibel H, Schlesier M, Peter HH (2002). Severe deficiency of switched memory B cells (CD27(+)IgM(-)IgD(-)) in subgroups of patients with common variable immunodeficiency: a new approach to classify a heterogeneous disease. Blood.

[B17] Penna G, Sozzani S, Adorini L (2001). Cutting edge: selective usage of chemokine receptors by plasmacytoid dendritic cells. J Immunol.

[B18] Bayry J, Lacroix-Desmazes S, Kazatchkine MD, Galicier L, Lepelletier Y, Webster D, Lévy Y, Eibl MM, Oksenhendler E, Hermine O, Kaveri SV (2004). Common variable immunodeficiency is associated with defective functions of dendritic cells. Blood.

[B19] Fayette J, Dubois B, Vandenabeele S, Bridon JM, Vanbervliet B, Durand I, Banchereau J, Caux C, Briere F (1997). Human dendritic cells skew isotype switching of CD40-activated naive B cells towards IgA1 and IgA2. J Exp Med.

[B20] Fayette J, Durand I, Bridon JM, Arpin C, Dubois B, Caux C, Liu YJ, Banchereau J, Briere F (1998). Dendritic cells enhance the differentiation of naive B cells into plasma cells in vitro. Scand J Immunol.

